# Neurofibfrome plexiforme cervical: à propos d’un cas

**DOI:** 10.11604/pamj.2018.30.41.14446

**Published:** 2018-05-17

**Authors:** Lamiae Bouimetarhan, Habib Bellamlih, Issam En-nafaa, Jamal El Fenni, Touria Amil, Bouchaib Radouane

**Affiliations:** 1 Service de Radiologie, Hôpital Militaire d’Instruction Mohammed V, Université Mohammed V, Rabat, Maroc

**Keywords:** Neurofibromatose, plexiforme, perinevre, IRM, Neurofibromatosis, plexiform, perineurium, MRI

## Abstract

Le neurofibrome plexiforme est une tumeur bénigne rare des nerfs périphériques aux dépens des cellules conjonctives du périnevre. Il est pathognomonique de la neurofibromatose de type 1 (NF1 ou maladie de Von Recklinghausen). L'IRM est d'une grande aide au diagnostic de cette pathologie. La confirmation anatomopathologique est parfois nécessaire en particulier en dehors d'un contexte évocateur d'une NF1. Nousrapportonsl'observation d'une petite fille atteinte de neurofibrome plexiforme cervical révélateur d'une neurofibromatose Type 1.

## Abstract (en)

Plexiform neurofibroma is a rare benign tumor of the peripheral nerves involving the conjunctiva cells of the perineurium. It is pathognomonic of neurofibromatosis type1 (NF1 or Von Recklinghausen disease). MRI is a great help in the diagnosis of this pathology. Anatomopathological confirmation is sometimes necessary, in particular in patients with no signs of NF1. We here report the case of a little girl with cervical plexiform neurofibroma revealing neurofibromatosis type 1.

## Introduction

Les neurofibromes sont des tumeurs bénignes qui se développent à partir des racines et des plexus des nerfs rachidiens. Ils peuvent être uni ou bilatéraux, parfois étagés, superficiels ou profonds. Les neurofibromes sont soit cutanés, diffus ou plexiformes. Les neurofibromes plexiformes correspondent morphologiquement à un segment plus ou moins long de dilatation tortueuse d'un nerf et de ses branches prenant un aspect de sac de vers. Les neurofibromes plexiformes sont pathognomoniques de la NF1. Ce sont des tumeurs généralement à croissance lente. Leur symptomatologie est variable fonction de leur topographie. Nous rapportons le cas d'une petite fille de 7ansqui consulte pour masse cervicale droite.

## Patient et observation

**Figure 1:**
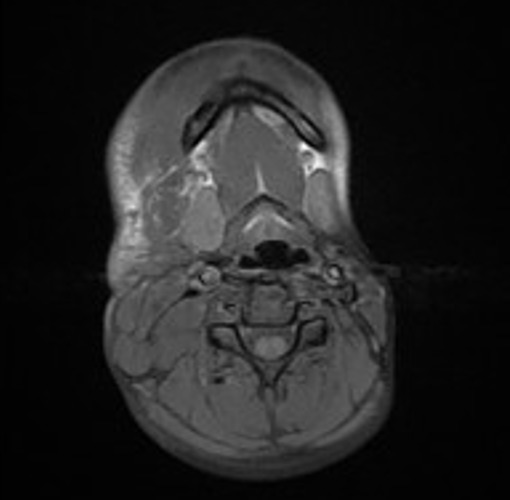
IRM cervicale, coupe axiale en séquence pondérée T1 sans injection de gadolinium : Processus latéro cervical droit en hypo signal T1

**Figure 2:**
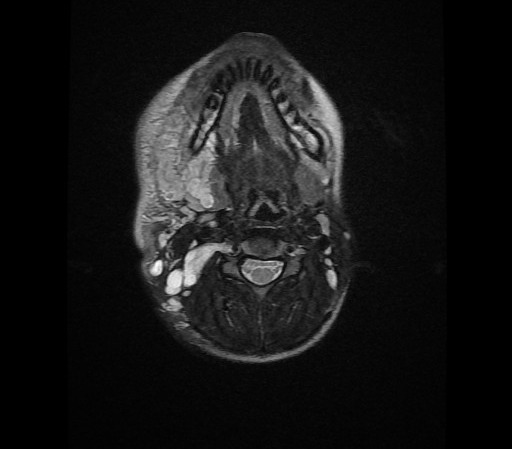
IRM cervicale, coupe axiale en séquence pondérée T2: processus lésionnel latéro cervical droit de contours lobulés en hypersignal T2 prenant naissance du foramen droit de C2-C3 (de la racine droite de C3) qui est élargit

**Figure 3:**
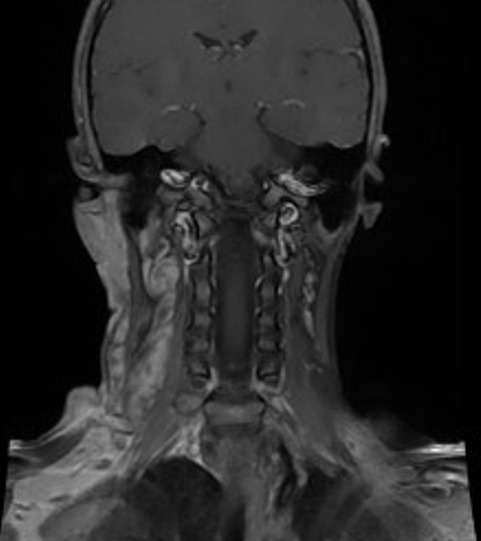
IRM cervicale,coupe coronale en séquence pondérée T1 après injection de gadolinium: la masse est rehaussée fortement après injection de gadolinium

**Figure 4:**
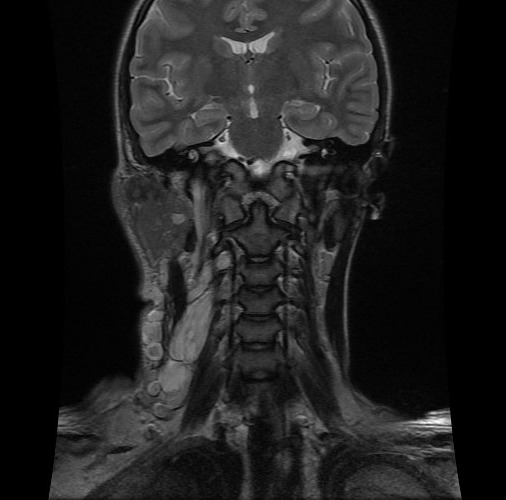
IRM cervicale, coupe coronale en séquence pondérée T2: processus lésionnel latéro cervical droit de contours lobulés en hypersignal T2 prenant naissance du foramen droit de C2-C3, infiltrant le muscle sterno cleido mastoïdien et étendu en bas au creux sus claviculaire

C'est une fille de 7 ans, ayant comme ATCD un père atteint de neurofibromatose type I, qui a consulté pour une masse latéro-cervicale droite avec des névralgies cervico-brachiales droites invalidantes. L'examen clinique trouve une masse de consistance molle indolore mal limitée sans signes inflammatoires en regard. Les aires ganglionnaires étaient libres et l'examen cutanérévèle la présence de multiples taches café au lait au niveau du dos et des membres (plus de 5) associé à des lentigines du creux axillaire. Devant ce tableau clinique très évocateur et les ATCD de la patiente le diagnostic de neurofibromatose type I a été évoqué. Une IRM cervicale aété réalisée en vu de caractériser la masse sus décrite et a révélé la présence d'un processus lésionnel latéro cervical droit de contours lobulés, hétérogène en hyposignal T1 (Figure 1), hypersignal T2 (Figure 2) rehaussé de façon hétérogène après injection de gadolinium (Figure 3) prenant naissance du foramen droit de C2-C3 (de la racine droite de C3) qu'il élargits'étend en péri vasculaire en englobent l'axe jugulo-carotidien puis infiltre les tissus mous en particulier les structures musculaires, la glande parotide et la glande sous maxillaire droite pour s'étendre ensuite en bas jusqu'à la région sus claviculaire (Figure 4). Le diagnostic de neurofibrome plexiforme de la racine droite de C3 sur NF1 a été retenu.

## Discussion

Le neurofibrome plexiforme est une tumeur bénigne rare des nerfs périphériques aux dépens des cellules conjonctives du périnevre.Son caractère non encapsulé explique l'infiltration diffuse des troncs nerveux adjacents, du tissu cellulo graisseux et du muscle.Il est souvent considéré comme pathognomonique de la neurofibromatose de type1 (NF1 ou maladie de Von Recklinghausen) et se voit beaucoup plus fréquemment que le schwannome au cours de la NF1 [1,2]. Le NFP appartient aux quatre types de neurofibromes rencontrés dansla NF1 selon la classification de la conférence de consensus de 1988 du National Institute of Health Development. Le diagnostic de la maladie repose sur la présence d'au moins deux des critères suivants [3,4]: au moins six taches café au lait de plus de 5mm avant la puberté et plus de 15mm après la puberté; Deux neurofibromes ou plus, un névrome plexiforme ou plus; Des taches lentigineuses de la région axillaire ou inguinale; Deux hamartomes de l'iris ou plus(nodules de Lisch),un gliome des voies optiques; Une lésion osseuse caractéristique (pseudarthrose d'un os long, dysplasie sphéno-orbitaire, cyphose cervicale). Il peut être observé des neurofibromes plexiformes isolés en dehors d'un contexte de NF1dont le diagnostic doit rester un diagnostic d'élimination. Il semblerait que certains de ces cas s'intègrent dans le cadre d'une neurofibromatose segmentaire (NF5) [5]. Ce sont des tumeurs généralement à croissance lente, cependant l'évolution de leur croissance est imprévisible. Une croissance rapide peut survenir au cours de la puberté ou de la grossesse sans régression spontanée. Ils peuvent être uni ou bilatéraux et siéger à différents niveaux ainsi leur symptomatologie est variable fonction de leur topographie. Le NFP se développe à partir des rameaux nerveux de la cinquième, septième ou neuvième paire crânienne au cours de la localisation cranio-faciales. Au niveau cervical, le neurofibrome ou le névrome plexiforme peut être à l'origine d'une compression médullaire ou peut s'étendre au plexus sympathique ou au plexus brachial engendrant un syndrome de Claude Bernard Horner ou une paralysie nerveuse périphérique [5,6]. Aux étages thoracique et abdominal ils posent un problème de diagnostic différentiel avec une tuberculose, un lymphome ou une sarcoïdose [1,5-7]. A l'étage pelvien ils simulent des adénopathies ou des abcès du psoas. L'imagerie est d'une grande aide au diagnostic permettant ainsi de caractériser les lésions en vu d'un diagnostic positif, de rechercher d'éventuelles lésions associées, d'évaluer le pronostic et de faire un suivi.

A l'échographie, les NP se traduisent par des masses lobulées tortueuses réalisant un aspect en grappe, hypo échogène, aux contours bien limités orientées le long de l'axe du tronc nerveux sur les coupes longitudinales et se caractérisant par le Target sign sur les coupes transversales avec un centre échogène et une périphérie hypo échogène. On peut observer des formations kystiques au sein de la masse ainsi qu'un renforcement postérieur rencontré dans 70% des cas. Le doppler couleur révèle différents types de 8; vascularisation modérée, centrale ou à prédominance périphérique. Certains peuvent être faiblement vascularisés [8]. L'étude tomodensitométrique révèle des lésions nodulaires, fusiformes ou en grappe, moins denses que le muscle (20-30UH) Cette densité basse est expliquée par la présence d'inclusions lipidiques au niveau des cellules de Schwann, d'adipocytes, d'une dégénérescence kystique et d'un stroma mixoide. Le comportement après injection est variable: prise de contraste homogène ou hétérogène [1]. Le NP se traduit sur les séquences d'Imagerie par résonnance magnétique qui constitue l'examen radiologique de référence, par un hypo signal relatif T1 par rapport au muscle, hyper signal T2 et lorsqu'il est volumineux, il peut renfermer un hypo signal central réalisant un aspect en cocarde caractéristique .Le rehaussement est variable: central, diffus, périphérique, en cible [2,5,9]. Le diagnostic deNFP est essentiellement anatomopathologique, en particulier en dehors du contexteévocateur de neurofibromatose de type I. Anciennement appelé névrome plexiforme ou tumeur royale, le NFP diffère des autres types de neurofibromes par l'importance de sa composante schwannienne [10]. Le risque de dégénérescence sarcomateuse justifie, chaque fois que cela est techniquement possible, l'exérèse de la lésion aussi complète que possible. Enfin, il convient de réaliser, au mieux par une équipe pluridisciplinaire, une surveillance clinique et radiologique de ces patients, au moins annuelle jusqu'à l'âge de dix ans, puis régulière, afin d'évaluer une éventuelle récidive ou une transformation maligne [5].

## Conclusion

Les neurofibromes plexiformes sont des tumeurs bénignes rares des nerfs périphériques.Ce sont des tumeurs pathognomoniques de la NF 1 qui est une pathologie multi systémique avec un polymorphisme clinique et radiologique. L'IRM constitue l'examen de choix dans l'exploration de cette pathologie.Elle apporte des arguments décisifs aussi bien pour le diagnostic positif, l'évaluation du pronostic et le suivi évolutif des lésions.

## Conflits d’intérêts

Les auteurs ne déclarent aucun conflit d'intérêts.
